# EUROLD: preliminary results of the ecological study on suicide and its associated socioeconomic variables in people over 85 in Europe

**DOI:** 10.1192/j.eurpsy.2023.777

**Published:** 2023-07-19

**Authors:** J. P. Carrasco Picazo, M. Rodríguez Ruzafa, G. Junquera Fernández, E. J. Aguilar

**Affiliations:** 1 Psychiatry, Hospital Clínico Universitario de Valencia, Valencia; 2 Psychiatry, Hospital Universitario Reina Sofía, Córdoba; 3 Psychiatry, CIBERSAM, Madrid; 4 Psychiatry, INCLIVA; 5 Psychiatry, Faculty of Medicine, Valencia, Spain

## Abstract

**Introduction:**

Approximately one person commits suicide every 40 seconds, resulting in more than 800,000 deaths per year worldwide. Regarding this phenomenon, it is necessary to highlight how suicide rates increase markedly with age. These reach their highest figures in people aged 85 years or older, and this increase is very worrying in certain geographical areas. Although there is extensive literature on the risk factors that influence at the individual level, the same cannot be said when the problem is analyzed at the population level.

**Objectives:**

The study aims to review the entire Eurostat database, relating suicide data from different European countries to any possible variables that may influence suicide. In this pilot phase, certain socioeconomic variables were chosen based on criteria of suitability and availability of the information provided, selecting data from 2015, as it was the most recent year in which most countries reported their data on suicide in people over 85 years of age.

**Methods:**

Firstly, a comparison was made of suicide rates in people over 85 years of age in relation to overall suicide rates in different European countries (suicide rate in people over 85 years of age divided by the total rate in the country). Secondly, socioeconomic variables that may be more strongly related to suicide in this age group in these European countries were studied. After calculating the conditional suicide rate in people over 85 years of age with respect to the overall suicide rate in each country (Fig. 1), Spearman correlations were performed between the conditional rates and different demographic variables, economic variables, social variables, and health variables.

**Results:**

Conditional suicide rates in people over 85 years of age show a marked difference between southern and northern European countries. In the correlational analysis, several significant associations were found. Suicide in those over 85 years of age was associated with economic variables (social deprivation, economic impossibility to buy new clothes, impossibility to dedicate money for personal matters and Gini coefficient), demographic (old-age dependency ratio) and health (self-perceived health). After performing a multivariate regression with the variables that were significant in the Spearman correlation, included the variables “old-age dependency ratio (X1)” and “economic impossibility to buy new clothes (X2),” with a value of R-square = 0.612 and a value of p < 0.01.

**Image:**

**Figure:**
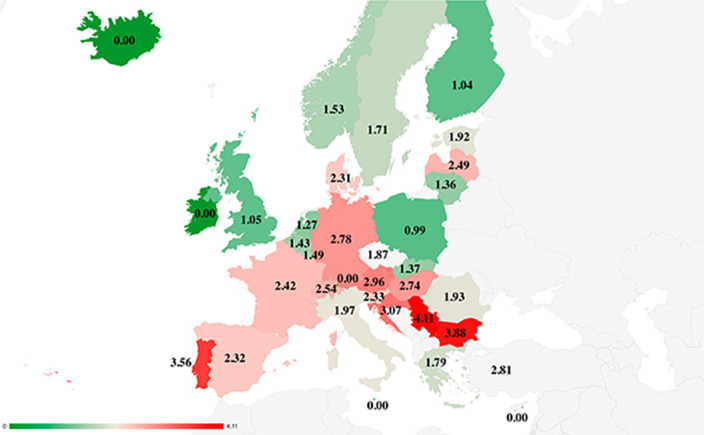

**Conclusions:**

The conclusions suggest that of the different variables studied, the great majority in which an association has been found belong to the field of economics, specifically poverty and economic inequality, and demographics, highlighting the old-age dependency ratio. Furthermore, marked north/south differences can be observed in the different European countries.

**Disclosure of Interest:**

None Declared

